# Altered Functional Connectivity of the Salience Network in Problematic Smartphone Users

**DOI:** 10.3389/fpsyt.2021.636730

**Published:** 2021-07-19

**Authors:** Jaeun Ahn, Deokjong Lee, Kee Namkoong, Young-Chul Jung

**Affiliations:** ^1^Psychiatry, National Health Insurance Service Ilsan Hospital, Goyang, South Korea; ^2^Institute of Behavioral Science in Medicine, Yonsei University College of Medicine, Seoul, South Korea; ^3^Psychiatry, Yongin Severance Hospital, Yonsei University College of Medicine, Yongin, South Korea; ^4^Psychiatry, Severance Hospital, Yonsei University College of Medicine, Seoul, South Korea

**Keywords:** probelmatic smartphone use, salience network, functional connectivity, fMRI, neuroimaging

## Abstract

Smartphones provide convenience in everyday life. Smartphones, however, can elicit adverse effects when used excessively. The purpose of this study was to examine the underlying neurobiological alterations that arise from problematic smartphone use. We performed resting state seed-based functional connectivity (FC) analysis of 44 problematic smartphone users and 54 healthy controls. This analysis assessed the salience, central executive, default mode, and affective networks. Compared to controls, problematic smartphone users showed enhanced FC within the salience network and between the salience and default mode network. Moreover, we observed decreased FC between the salience and central executive network in problematic smartphone users, compared to controls. These results imply that problematic smartphone use is associated with aberrant FC in key brain networks. Our results suggest that changes in FC of key networks centered around the salience network might be associated with problematic smartphone use.

## Introduction

For the past two decades, smartphones have radically changed human lives, becoming ubiquitous in everyday life (1, 2). Smartphones have brought about changes to various areas of life, such as productivity, information seeking, social information and interaction, diversion, relaxation, entertainment, monetary compensation, and personal status (3). However, with greater integration of smartphones into daily lives, concerns for psychological and behavioral dysfunction due to problematic smartphone use have begun to accumulate (4, 5).

The clinical characteristics of problematic smartphone use share conceptual similarities with typical addictive disorders (6). Lin et al. have suggested a diagnostic standard for smartphone addiction, with core symptoms of “impaired control” and withdrawal (7). According to the Interaction of Person-Affect-Cognition-Execution model, which lays the theoretical framework for addictive behaviors, an association between cue-reactivity/craving and diminished inhibitory control contribute to the development of addictive behaviors (8, 9). The frontoinsular cortex, which acts as a mediator between the limbic and prefrontal-striatal system, and an imbalance between hyperactive involvement of limbic structures and hypoactive involvement of prefrontal-striatal circuits are thought to play crucial roles in the pathophysiology of addictive behaviors (8, 9). While numerous attempts have been made to identify neural correlates undergirding behavioral addiction, most studies have been published on gambling and Internet gaming disorder; neural correlates responsible for problematic smartphone usage remain largely unknown.

Resting-state functional magnetic resonance imaging is a powerful tool that can be utilized to investigate correlates related to various neurological and psychiatric disorders. The salience network, central executive network, default mode network, and affective network play key functions in the human brain (10, 11). The salience network, comprising nodes in the right frontoinsular cortex and anterior cingulate cortex, facilitates orientation to stimuli and allocates attention (12). The central executive network, which is responsible for goal-directed behavior involving decision making, comprises the dorsolateral prefrontal cortex and posterior parietal cortex (12). The default mode network, which is responsible for stimulus-independent thought processes, comprises the medial prefrontal cortex, the rostral parts of the anterior cingulate cortex, the precuneus, and the posterior cingulate cortex (13). Lastly, the affective network, which is relevant for emotion perception and regulation, comprises a set of interconnected neural structures in the amygdala and subgenual anterior cingulate cortex (14, 15).

Research into changes in intrinsic connectivity networks in behavioral addiction has primarily been limited to Internet gaming disorders and has revealed aberrant functional connectivity (FC) between and within intrinsic connectivity networks in the brain (16–18). In Internet gaming disorder, deficient modulation of central executive network activity versus default mode network activity by the salience network has been suggested as a neurobiological mechanism in the maintenance of addictive behaviors (16). Furthermore, altered FC to the frontal lobe over the amygdala has been found to contribute to vulnerability to Internet gaming disorder (19). Meanwhile, a few neuroimaging studies on problematic smartphone use have also suggested changes in FC in brain regions related with cognitive control and emotional processing: One study reported altered neural deactivation of the prefrontal and cingulate cortex during facial emotion processing in problematic smartphone users, compared to controls (20). These results suggest that problematic smartphone use may affect cognitive control during emotional processing *via* altered integrity of functional brain networks. Another study reported that adolescent problematic smartphone users had reduced FC in brain regions related to cognitive control (21). Although these studies provide some insights into the neurobiological basis for problematic smartphone use, relatively few neuroimaging studies have focused on underlying neural correlates responsible for problematic smartphone use.

Thus, we aimed to identify changes in intrinsic connectivity networks in problematic smartphone use. We investigated alterations in FC among core intrinsic connectivity networks (salience network, central executive network, default mode network, and affective network) in problematic smartphone users based on regions of interest in these core networks.

## Materials and Methods

### Participants

Ninety-eight subjects participated in this study: 29 males with excessive smartphone use, 15 females with excessive smartphone use, 32 healthy males, and 22 healthy females. All subjects were right-handed and between 16 and 54 years of age (mean: 23.6 ± 4.8 years). All participants were administered the Structured Clinical Interview from the Diagnostic and Statistical Manual of Mental Disorders, Fourth Edition to evaluate major psychiatric illness (22). The Korean version of the Wechsler Adult Intelligence Scale IV was used to assess intelligence quotient (23). Exclusion criteria for all subjects were major psychiatric disorder, intellectual disability, neurological or medical illness, and contraindications on magnetic resonance imaging (MRI) scan. None of the subjects included in this study received psychiatric treatment, including psychopharmacology.

### Psychometric Measures

The Smartphone Addiction Proneness Scale (SAPS) test, developed by the Korean National Information Society Agency to assess problematic smartphone use (24), consists of 15 questions and includes the following four subscales: disturbance of adaptive functions, virtual life orientation, withdrawal, and tolerance. Subjects with the following SAPS scores were classified as high-risk smartphone users: (a) total SAPS score of ≥44 and (b) disturbance of adaptive functions, withdrawal, and tolerance subscale scores of ≥15, ≥13, and ≥13, respectively. In this study, subjects with the following SAPS scores were classified as potentially at-risk smartphone users: (a) total SAPS score of ≥40 and ≤ 43 or (b) disturbance of adaptive functions subscale score of ≥14. The high-risk or potentially at-risk users were classified as problematic smartphone users. The Cronbach's alpha of the SAPS was 0.932 in the present sample.

The Internet Addiction Test (IAT) was administered to assess Internet addiction status (25), with a Cronbach's alpha value of 0.933 in the present sample. The Barratt Impulsiveness Scale version 11 (BIS-11) was administered to test impulsivity (26), with a Cronbach's alpha in the present sample of 0.800. To evaluate comorbid psychiatric conditions of depression, anxiety, and alcohol use disorder, all subjects were administered the Beck Depression Inventory (BDI, Cronbach's alpha = 0.790), Beck Anxiety Inventory (BAI, Cronbach's alpha = 0.796), and Alcohol Use Disorder Identification Tests (AUDIT, Cronbach's alpha = 0.782), respectively.

### FC Analysis

Brain MRI data were acquired using a 3T MRI scanner (Magnetom; Siemens, Munich, Germany) equipped with an eight-channel head coil. The structured MRI data were obtained through a T1-weighted spoiled gradient echo sequence (echo time = 2.19 ms, repetition time = 1,780 ms, flip angle = 9°, field of view = 256 mm, matrix = 256 × 256, transverse slice thickness = 1 mm). The functional MRI data were obtained through a single-shot T2-weighted gradient echo planar pulse sequence (echo time = 30 ms, repetition time = 2,500 ms, flip angle = 90°, field of view = 240 mm, matrix = 64 × 64, slice thickness = 3.5 mm). During the acquisition of functional MRI data, subjects were instructed to look at a white cross in the center of a black background for 6 min without any cognitive, lingual, or motor activities.

Imaging data were processed using a Microsoft Windows platform running MATLAB version 9.3 (R2020a) (The MathWorks Inc, Natick, MA, USA) and the MATLAB-based CONN-fMRI Functional Connectivity toolbox, version 19.c (Cognitive and Affective Neuroscience Laboratory, Massachusetts Institute of Technology, Cambridge, MA, USA). The default CONN preprocessing pipeline was applied. To correct motion across volumes, functional images were aligned to the first volume using a least-square minimization and a six-parameter rigid body spatial transformation. Unwarping and slice-timing correction were also applied.

Next, we ran the ART-based automatic outlier detection for later scrubbing. Specifically, functional volumes were deemed outliers if their signal intensity deviated more than five standard deviations from the mean signal intensity of the whole series or showed evidence of displacement superior to 0.9 mm in relation to the preceding volume. Subjects for whom more than 15% of frames were censored for scrubbing were excluded: all subjects were included in the FC analysis because none met these exclusion criteria. Both functional and structural images were then subjected to gray and white matter and cerebrospinal fluid segmentation, and a bias correction was performed to remove varying intensity differences across images.

Finally, structural and functional data were spatially normalized in parallel through non-linear transformations to the Montreal Neurological Institute space. Images were re-sliced to a 2-mm isotropic resolution and smoothed with an 8-mm full-width at half-maximum (FWHM) isotropic Gaussian kernel. After preprocessing, imaging data were denoised from residual movement and physiological noise (i.e., respiration, cardiac pulsations, slow involuntary head position motion, or “spike-like” movements) (27). Specifically, the denoising steps included a temporal de-spiking, regressing out confounding factors (i.e., BOLD signal small ramping effects at the beginning of each session, six rigid body realignment parameters and their first order derivatives), an anatomical component-based noise correction method (aCompCor, which reduces physiological and movement noise), the ART scrubbing protocol, linear detrending to remove linear signal drift, and band-pass filtering to restrict the analysis to a range of frequencies of interest (0.008–0.09 Hz).

Seed-to-voxel FC maps for each subject were constructed using the CONN-fMRI toolbox 19.c (http://www.nitrc.org/projects/conn). While configuring individual seed-to-voxel FC maps, movement parameters for each subject were preserved as confounders within the general linear model. For four networks, the following spherical seeds with a 6-mm radius were selected: the dorsal anterior cingulate cortex (6 45 9) (28) and the anterior frontoinsular cortex (left anterior frontoinsular cortex: −45 35 9; right anterior frontoinsular cortex: 45 3 15) (29) of the salience network; the bilateral frontal eye field (left frontal eye field: −24 −15 66; right frontal eye field: 28 −10 58) (29) and bilateral dorsolateral prefrontal cortex (left dorsolateral prefrontal cortex: −40 18 24; right dorsolateral prefrontal cortex: 40 18 24) (30) of the central executive network; the posterior cingulate gyrus (−5 −49 −40) (31), the medial prefrontal cortex (−1 47 −4) (31), and the bilateral rostral anterior cingulate cortex (left rostral anterior cingulate cortex: −5 34 28; right rostral anterior cingulate cortex: 5 34 28) (32) of the default mode network; and the bilateral amygdala (left amygdala: −24 −4 −16; right amygdala: 24 −4 −16) (33) and bilateral subgenual anterior cingulate cortex (left subgenual anterior cingulate cortex: −5 25 −10; right subgenual anterior cingulate cortex: 5 25 −10) (32) of the affective network were selected as regions of interest (ROIs). All seed regions were built using the MarsBaR toolbox in SPM to create 6-mm spherical ROI images. Signals from white matter and ventricular regions were also eliminated through linear regression (34). To reduce artifacts caused by head motion, estimated subject-motion parameters (35) implemented in CONN's default denoising guideline were applied to the linear regression model. Correlation coefficients were estimated and converted to z-values using Fisher's r-to-z transformation for the calculation of FC strengths. Afterwards, FC strength estimates were compared between groups using an analysis of covariance (ANCOVA) on each voxel, after controlling for age and sex. All imaging analyses were corrected for multiple comparisons using a combination of voxel-level thresholds (*p* < 0.001) and cluster extent threshold false discovery rate correction (*p* < 0.05).

### Statistical Analysis

Statistical analyses were conducted using the Statistical Package for the Social Sciences (SPSS) version 25.0 (SPSS Inc., Chicago, IL, USA). Differences with *p*-values < 0.05 were considered statistically significant. To compare demographic and psychological features, we employed independent Student's *t*-tests and χ^2^ tests. We conducted correlation analyses to verify that FC strengths were correlated with clinical variables (smartphone usage times, SAPS score, and BIS score). In subsequent partial correlation analyses, parameters related to comorbidity conditions (age and gender) were added as covariates.

### Ethics

This study was carried out in accordance with the latest version of the Declaration of Helsinki, and under the guidelines for human subject research established by the Institutional Review Board at Yonsei University. All protocols for this study were approved by the Institutional Review Board at Severance Hospital, Yonsei University. Written informed consent was obtained from all participants before enrollment.

## Results

### Participant Characteristics

Problematic smartphone users and controls did not significantly differ with respect to age, sex, full scale IQ, or AUDIT scores (Table 1). Psychometric self-repost scales showed significant group differences in SAPS (*p* < 0.001), IAT (*p* < 0.001), BDI (*p* = 0.013), BAI (*p* = 0.001), and BIS (*p* = 0.002) scores. Problematic smartphone users spent significantly more time daily using their smartphones than the control group (*p* < 0.001).

**Table 1:** Demographics and clinical characteristics of study participants.

	**Excessive smartphone users** **(*n* = 44) Mean (SD)**	**Healthy controls** **(*n* = 54) Mean (SD)**	**Test**	***df***	**Test**
Age (years)	24.6 (6.1)	22.7 (3.3)	t = 1.871	96	t = 1.871
Sex (male), number (%)	29 (47.5%)	32 (52.5%)	χ^2^ = 0.456	1	χ^2^ = 0.456
Full-scale IQ^a^	110.5 (12.0)	109.7 (10.7)	t = 0.341	96	t = 0.341
SAPS	45.0 (4.7)	29.4 (6.3)	t = 13.983	95.299	t = 13.577
Disturbance of adaptive functions	15.3 (1.8)	9.6 (2.4)	t =13.454	95.391	t =13.070
Virtual life orientation	4.2 (1.3)	2.8 (1.0)	t = 6.055	96	t = 6.055
Withdrawal	11.8 (2.2)	8.1 (2.5)	t = 7.749	96	t = 7.749
Tolerance	13.6 (1.5)	8.9 (2.7)	t = 10.875	84.497	t = 10.279
IAT	48.8 (13.6)	36.8 (17.7)	t = 3.693	96	t = 3.693
BIS	55.5 (8.8)	49.8 (8.7)	t = 3.207	96	t = 3.207
BDI	8.7 (4.6)	6.2 (5.0)	t = 2.531	96	t = 2.531
BAI	8.5 (5.9)	5.0 (4.4)	t = 3.239	78.262	t = 3.333
AUDIT	9.6 (5.3)	7.7 (5.1)	t = 1.739	96	t = 1.739
Duration of smartphone use per day (hours)	6.9 (2.1)	2.6 (1.2)	t = 12.147	65.910	t = 12.783

*AUDIT, alcohol use disorder identification test; BAI, Beck anxiety inventory; BDI, Beck depression inventory; BIS, Barratt impulsivity scale; IAT, Internet addiction test; IQ, intelligence quotient; SAPS, smartphone addiction proneness scale. SD, standard deviation*.

a *IQ was assessed using the Wechsler Adult Intelligence Scale*.

### FC Analysis Results

#### Salience Network

**Figure 1:**
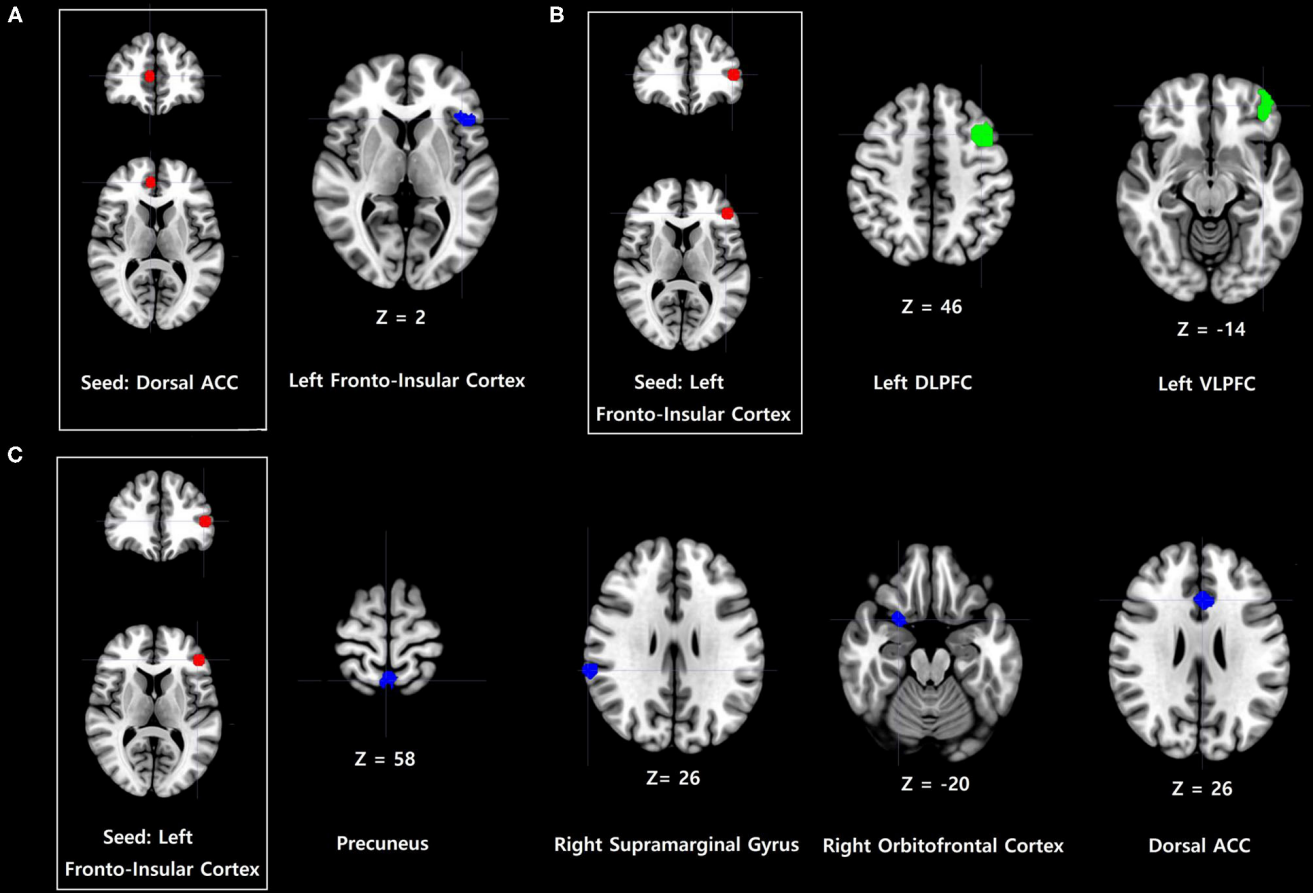
Increases and decreases in dorsal anterior cingulate cortex (dorsal ACC) and frontoinsular cortex-based functional connectivity (FC) in problematic smartphone users, compared to controls. All imaging analyses were corrected for multiple comparisons using a combination of voxel-level thresholds (*p* < 0.001) and cluster extent threshold false discovery rate correction (*p* < 0.05). **(A)** Dorsal ACC-based FC was enhanced with the left anterior frontoinsular cortex in problematic smartphone users, compared to controls. **(B)** Left frontoinsular cortex-based FC was decreased with left ventrolateral prefrontal cortex (VLPFC) and left dorsolateral prefrontal cortex (DLPFC) in problematic smartphone users, compared to controls. **(C)** Left frontoinsular cortex-based FC was enhanced with precuneus, right supramarginal gyrus, right orbitofrontal cortex and dorsal ACC.

Problematic smartphone users showed stronger FC between the dorsal anterior cingulate cortex and anterior frontoinsular cortex, as well as anterior frontoinsular cortex-based FC with the precuneus, supramarginal gyrus, orbitofrontal cortex, and dorsal anterior cingulate cortex, relative to control users (Table 2, Figure 1). In contrast, problematic smartphone users demonstrated weaker anterior frontoinsular cortex-based FC with the dorsolateral prefrontal cortex, and ventrolateral prefrontal cortex than control users.

**Table 2:** Whole-brain seed-based functional connectivity analysis results.

	**Region**	**BA**	**kE**	**Tmax**	**x**	**y**	**z**	
**Salience network - dorsal ACC**								
Excessive smartphone users > control	Left	Anterior FIC	48	209	4.23	−40	24	2
**Salience network – anterior FIC**								
Excessive smartphone users > control	Left	Precuneus	5	207	3.82	−4	−42	58
	Right	Supramarginal gyrus	5	169	4.5	64	−34	26
	Right	Orbitofrontal cortex	34	266	5.3	24	8	−20
	Left	Dorsal ACC	24	354	4.76	−6	20	26
Control > excessive smartphone users	Left	DLPFC	9	190	4.01	−16	30	50
	Left	VLPFC	47	361	4.59	−38	38	−12
**Central executive network - DLPFC**								
Excessive smartphone users > control	Left	Postcentral gyrus	3	188	4.11	−66	−10	32
**Default mode network - rostal ACC**								
Excessive smartphone users > control	Left	Anterior FIC	48	169	4.31	−26	20	12
**Affective network - Amygdala**								
Excessive smartphone users > control	Left	DLPFC	9	563	5.25	−34	12	46
**Affective network - subgenual ACC**								
Control > excessive smartphone users	Left	Lingual gyrus	18	233	4.4	−26	−84	−14

*ACC, anterior cingulate cortex; DLPFC, dorsolateral prefrontal cortex; FIC, frontoinsular cortex; VLPFC, ventrolateral prefrontal cortex. Brain regions showing a significant difference in functional connectivity between groups*.

#### Central Executive Network

**Figure 2:**
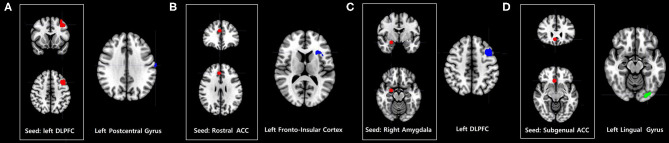
Increases and decreases in seed-based FC among problematic smartphone users, compared to controls. All imaging analyses were corrected for multiple comparisons using a combination of voxel-level thresholds (*p* < 0.001) and cluster extent threshold false discovery rate correction (*p* < 0.05). **(A–C)** Brain regions with enhanced FC in problematic smartphone users, compared to controls. **(A)** Left dorsolateral prefrontal cortex (DLPFC)-based FC was increased with the left postcentral gyrus. **(B)** Right rostral anterior cingulate cortex (ACC)-based FC was increased with the left anterior frontoinsular cortex. **(C)** Right amygdala-based FC was increased with DLPFC. **(D)** Brain regions with decreased FC in problematic smartphone users, compared to controls. The right subgenual ACC and left lingual gyrus show differences in functional connectivity with right subgenual ACC-seeded analysis between the groups.

Problematic smartphone users exhibited stronger dorsolateral prefrontal cortex FC with the left precentral gyrus than control users (Figure 2).

#### Default Mode Network

Problematic smartphone users showed greater right rostral anterior cingulate cortex FC with the anterior frontoinsular cortex than control users.

#### Affective Network

Compared to the control group, problematic smartphone users exhibited stronger FC between the amygdala and dorsolateral prefrontal cortex. Problematic smartphone users also showed weaker FC between the right subgenual anterior cingulate cortex and lingual gyrus.

### Correlation Between FC Strengths and Psychometric Measures

We found no significant correlations between FC strengths and clinical variables (smartphone usage times, SAPS score, and BIS score).

## Discussion

**Figure 3:**
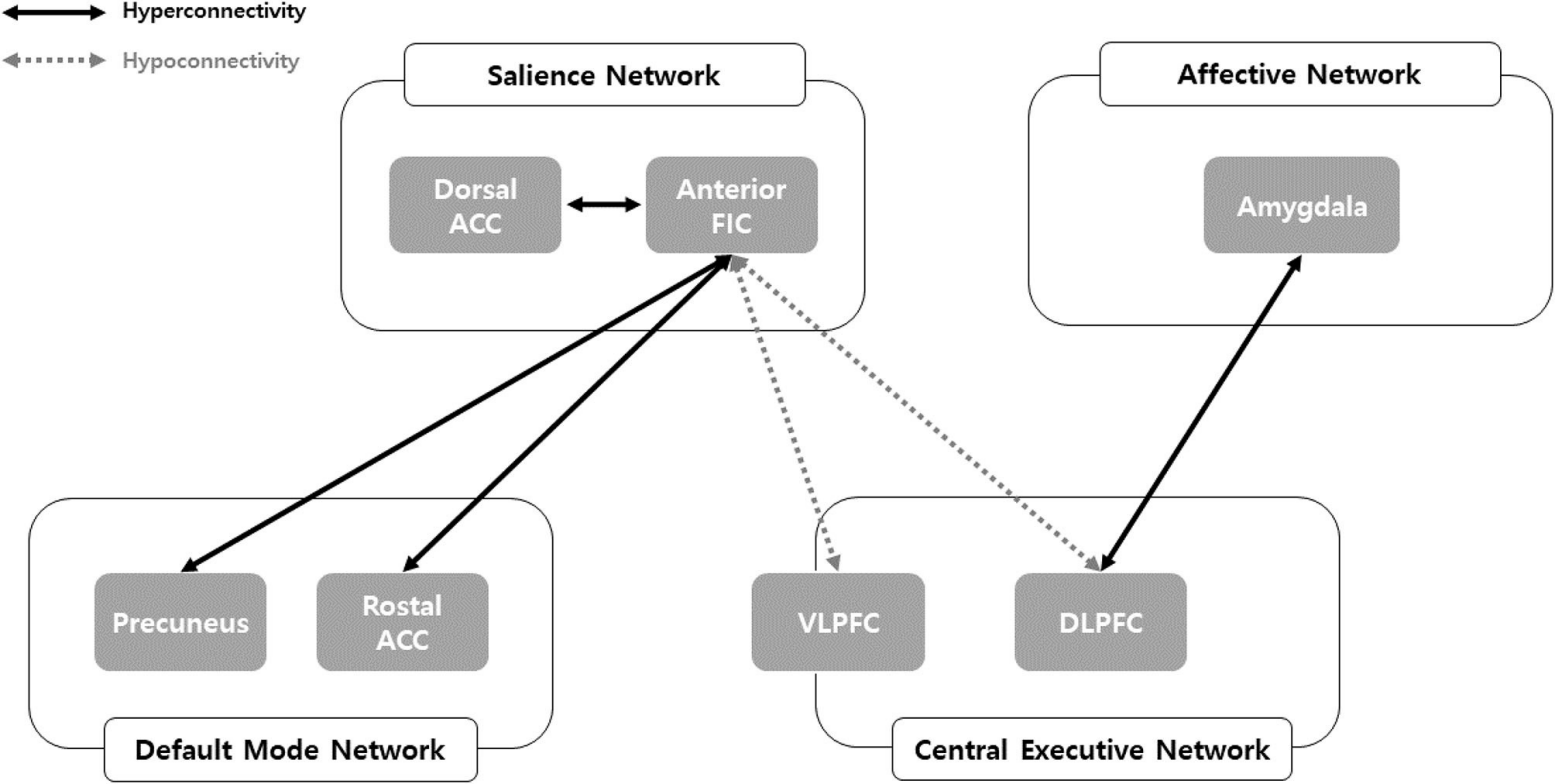
Network model of problematic smartphone users. ACC, anterior cingulate cortex; DLPFC, dorsolateral prefrontal cortex; FIC, frontoinsular cortex; VLPFC, ventolateral prefrontal cortex.

Consistent with our hypothesis, we comprehensively identified FC changes in the salience, central executive, default mode, and affective networks among problematic smartphone users (Figure 3). We observed enhanced FC within the salience network and between the default mode network and salience network in problematic smartphone users, compared to controls. Meanwhile, we noted decreased FC between the salience network and central executive network among problematic smartphone users. Overall, neurobiological changes in FC of key networks centered around the salience network were observed in problematic smartphone users.

Enhanced FC within the salience network was observed among problematic smartphone users. In particular, we noted enhanced FC between the anterior frontoinsular cortex and dorsal anterior cingulate cortex during the resting state and enhanced FC between the anterior frontoinsular cortex and supramarginal gyrus. The salience network reacts to degrees of subjective salience and acts as a switch between the default mode network and the central executive network (10, 36). The anterior frontoinsular cortex and dorsal anterior cingulate cortex are the two key nodes of the salience network (10); the supramarginal gyrus is also known as a salience network node (37). The anterior frontoinsular cortex facilitates bottom-up detection of salient events and modulates access to attention and working memory resources. Strong neural functional coupling between the anterior frontoinsular cortex and dorsal anterior cingulate cortex has been shown to facilitate rapid access to the motor system (10). Enhanced interaction within the salience network in problematic smartphone users may represent enhanced salience for cues related to smartphones and may provoke more frequent use of smartphones in problematic users.

Our data highlighted enhanced FC between the salience network and default mode network in problematic smartphone users, as enhanced FC was found between the rostral anterior cingulate cortex and anterior frontoinsular cortex, as well as between the precuneus and anterior frontoinsular cortex. The rostral anterior cingulate cortex and precuneus are critical hubs of the default mode network (38), and enhanced FC between the salience and default mode networks in problematic smartphone users suggests decreased engagement of executive control or reflective system (39). Our findings are consistent with previous studies that reported enhanced FC between the frontoinsular cortex and default mode network in nicotine-dependent smokers (40) and Individuals with Internet gaming disorder (17). Aberrant FC between the frontoinsular cortex and default mode network is known to be involved in development and maintenance of addiction (41). Altogether, these results suggest a need to consider changes in resting state FC in problematic smartphone users as indicative of behavioral addiction. Additionally, we also noted enhanced FC between the anterior frontoinsular cortex and orbitofrontal cortex in problematic smartphone users. The orbitofrontal cortex is a major area of motivation, drive, and salience evaluation and has been found to be impaired in drug addiction (42) and behavioral addiction (43–45). Overall, our results are consistent with viewing problematic smartphone use from the perspective of behavioral addiction.

Interestingly, we recorded reduced FC between the salience network and central executive network, observing reduced FC between the anterior frontoinsular cortex and dorsolateral prefrontal cortex and between the anterior frontoinsular cortex and ventrolateral prefrontal cortex in problematic smartphone users, compared to healthy controls. The dorsolateral prefrontal cortex is a critical node of the central executive network (10), and the ventrolateral prefrontal cortex, which coactivates with the central executive network, is known to be involved in numerous cognitive operations (43). In a previous study of altered core brain network interactions in adolescents with Internet gaming disorder, abnormal functional and structural connections between the salience network and central executive network were deemed to be mediators of impaired cognitive control in adolescents with Internet gaming disorder (41). Also, one previous study proposed weakened cognitive control, which is related to function of the central executive network, as an important neurobiological basis of Internet gaming disorder (43). When using smartphones, individuals need to find a balance between the bottom-up smartphone-related salient stimuli and top-down inhibitory control of excessive usage, which negatively affects daily function. The reduced FC between the salience network and central executive network in problematic smartphone users observed in this study may suggest that these individuals are unable to control their smartphone usage and thus succumb to the temptation of pleasure from overuse.

Also noteworthy is that we were able to identify enhanced FC between the affective network and salience network and between the affective network and central executive network. We observed enhanced FC between the amygdala and dorsolateral prefrontal cortex during the resting state in problematic smartphone users. The amygdala is a key area responsible for generating and processing emotion that is involved in bottom-up attention to emotional stimuli (44, 44), and researchers have reported that levels of impulsivity were positively correlated with amygdala-dorsolateral prefrontal cortex connectivity (46). These results may indicate that aberrant FC between the affective network and central executive network might contribute to impulsivity in problematic smartphone users.

Lastly, we observed aberrant FC in brain regions responsible for sensory processing (e.g., the postcentral gyrus and lingual gyrus). Previous studies have demonstrated that patients with Internet gaming disorder and problematic smartphone users show aberrant FC in brain regions responsible for sensory processing (44, 45). Taken together, aberrant FC in brain regions responsible for sensory processing may reflect “bottom-up” neural processing in problematic smartphone users.

We acknowledge that this study has several shortcomings. Firstly, this study was designed as a cross-sectional analysis and thus limited in identifying causal relationships between changes in resting state FC and problematic smartphone use. Secondly, this study was limited in that problematic smartphone usage was evaluated only by self-reporting questionnaires and clinical interviews. Future studies with more precise measures of smartphone usage patterns are needed. Third, resting-state scan time was relatively short. Sufficient scan length can improve reliability and enable rigorous censoring for motion correction (46). Lastly, the age distribution of the subjects enrolled in this study was relatively large. As smartphone usage time was evaluated via self-reports, differences in smartphone usage time and smartphone usage pattern across different age groups might not be accurately reflected in the study. These limitations might account for why we found no significant correlation between FC strength and clinical variables. When related research is conducted in the future, stratification analysis according to age will be required.

Despite these limitations, we identified changes in underlying neural correlates of problematic smartphone users by analyzing key networks, including the salience, central executive, default mode, and affective networks, from the perspective of behavioral addiction. Overall, our results from multiple network analysis suggests that neurobiological changes centered around the salience network are associated with problematic smartphone use.

## Data Availability Statement

The completely anonymized raw data supporting the conclusions of this article will be made available by the authors upon request to the corresponding author, without undue reservation.

## Ethics Statement

The studies involving human participants were reviewed and approved by Institutional Review Board at Yonsei University. The patients/participants provided their written informed consent to participate in this study.

## Author Contributions

DL and Y-CJ conceived and designed the study. DL recruited participants. JA analyzed data and drafted the manuscript. KN and Y-CJ provided critical revision of the manuscript and important intellectual content. All authors had full access to all data in the study, take responsibility for the integrity of the data and the accuracy of the data analysis, and critically reviewed and approved the final version of this manuscript for publication.

## Conflict of Interest

The authors declare that the research was conducted in the absence of any commercial or financial relationships that could be construed as a potential conflict of interest.
